# Research progress on the correlation between MRI and impairment caused by cerebral small vessel disease: A review

**DOI:** 10.1097/MD.0000000000035389

**Published:** 2023-10-06

**Authors:** Yang Wang, Zhirong Liu

**Affiliations:** a Department of Neurology, Xijing Hospital, Fourth Military Medical University, Xi’an, China; b Department of Neurology, 980th Hospital of PLA Joint Logistical Support Force (Bethune International Peace Hospital), Shijiazhuang, China.

**Keywords:** cerebral small vessel disease, cognitive impairment, gait impairment, magnetic resonance imaging, risk factor

## Abstract

Cerebral small vessel disease (CSVD) is a chronic global brain disease mainly involving small blood vessels in the brain. The disease can be gradually aggravated with the increase of age, so it is the primary cause of brain dysfunction in the elderly. With the increasing aging of the world population and the high incidence of cerebrovascular risk factors, the incidence of CSVD is increasing day by day. CSVD is characterized by insidious onset, slow progression, diverse clinical manifestations, and difficult early diagnosis. CSVD can lead to cognitive impairment, gait impairment, affective impairment, and so on. however, it has not received enough attention from researchers in the past. In recent years, some studies have shown that CSVD patients have a high proportion of related impairment, which seriously affect patients daily life and social functions. Currently, no clear preventive measures or treatments exist to improve the condition. With the development of magnetic resonance imaging, CSVD has become more and more recognized and the detection rate has gradually improved. This paper reviews the research progress of magnetic resonance imaging and cognitive impairment, gait impairment, affective impairment, urination disorder, swallowing disorder, and other disorders to provide a useful reference for the early diagnosis and treatment of CSVD and expand new ideas.

## 1. Introduction

Cerebral small vessel disease (CSVD) is a clinical disorder that affects the structure and function of intracranial arterioles, venules, and capillaries.^[1]^ CSVD is characterized by insidious onset, slow development, complex clinical manifestations, and difficult early diagnosis. It can gradually develop into cognitive impairment, physical disability and emotional changes over a long period. It develops in stages and presents a progressive course of the disease. CSVD is particularly common in people over 60 years old, and the incidence of CSVD in people over 90 years old is nearly 100%.^[2]^ With the rapid aging of the population and the high incidence of CSVD risk factors such as diabetes and hypertension, more and more people are suffering from CSVD. The total number and volume of CSVDS may be related to a variety of disorders such as cognitive impairment, gait impairment and affective disorder,^[3]^ which are the main reasons for the decline in the living ability of the elderly. Recently, the relationship between CSVD and cognitive impairment has become one of the research hotspots.

According to 1 clinical trial,^[4]^ about 80% of the elderly over 65 years old have clinical or neuroimaging manifestations of CSVD. Magnetic resonance imaging (MRI) is currently the most reliable imaging diagnosis method, which can guide the early detection, diagnosis, and treatment of CSVD. Typical MRI features include recent subcortical infarction, (also known as lacunar infarction), lacunar white matter hyperintensity (WMH), perivascular space (PVS), cerebral microhemorrhage (CMB), and cerebral atrophy, which may be present alone or in several groups together. Imaging changes of CSVD are correlated with clinical manifestations to varying degrees, which can lay an important foundation for the potential pathogenesis of CSVD and the diagnosis of modifiable risk factors. Therefore, in this review, we used PubMed to search the literature on MRI, imaging markers, CSVD, and cognitive, gait, affective, urination, and swallowing impairment, etc, then summarized the characteristics of different MRIs for CSVD-related disorders in order to provide useful references for the early diagnosis and treatment of CSVD. This study obtained ethical approval from the Medical Ethics Committee of the Xijing Hospital of Fourth Military Medical University (No: KY20232227-F-1).

## 2. Cognitive impairment and CSVD

### 2.1. Overview of cognitive impairment

Cognitive function refers to the process of the human brain receiving external information and transforming it into internal mental activities of acquiring knowledge or applying knowledge, including memory, executive function, language function, visuospatial, and structural ability, application and other cognitive fields. Vascular cognitive impairment (VCI) is a disorder characterized by cognitive impairment caused by vascular factors, ranging from mild VCI to vascular dementia. CSVD is the most common lesion that causes VCI.^[5]^ About half of all cases of vascular dementia are caused by CSVD.^[6]^ Studies have shown that 30% to 64% of CSVD patients are complicated with varying degrees of cognitive impairment,^[7,8]^ and there are certain differences in cognitive impairment among patients with different types of CSVD. CSVD lesions can accumulate in the brain parenchyma over time, thus increasing the risk of cognitive impairment. CSVD-related cognitive impairment is mainly manifested as attentional and executive dysfunction, and even affects the major areas of cognitive function, with more severe impairment in memory, attention, numeracy and verbal fluency, but less severe impairment in orientation.^[9]^

### 2.2. Risk factors for cognitive impairment in CSVD

The cognitive impairment of CSVD is affected by many factors such as poor lifestyle and chronic disease. Studies have shown that with the increase in age, the detection rate of CSVD and cognitive impairment increases.^[10,11]^ Lacunar infarction is an important predictor of cognitive impairment. Studies have shown that the impairment of cognitive function caused by lacunar infarction is mainly executive function.^[12]^ Moreover, there is a dose-dependent relationship between the number of lacunar infarctions and the degree of cognitive impairment, and even a small lacunar infarction will cause a certain degree of cognitive impairment.^[13,14]^ Different sites of lacunar infarction can cause differences in the manifestation of cognitive impairment.^[15]^ Qin et al^[16]^ found that CSVD patients with lacunar infarction of the thalamus have more total infarcts and lower connection density of the neural network, and the overall execution efficiency of the patients is significantly reduced, but the local execution efficiency is not significantly reduced. A meta-analysis^[17]^ has also shown that cognitive impairment caused by lacunar infarction is nonselective and may affect all major areas of cognitive function.

CSVD with WMH as the main imaging manifestation is associated with the decline of various cognitive functions, especially the decline of information processing speed and executive function.^[18]^ However, there are still some conflicts regarding WMH associated with cognitive and executive decline. Recently, to understand the relationship between WMH and cognition further, Hairu R et al^[19]^ performed a clinical trial including 26 patients with cognitive impairment caused by Alzheimer Disease. After systematic evaluation and measurement of WMH volumes, they found that WMH volume is positively associated with cognitive impairmentbased on the unadjusted data, but the correlation with executive function decline was only observed after adjustment for age. In contrast, another study focusing on the transient ischemic attack and minor stroke patients demonstrated the volume of WHM is related to the decline of executive function, and the larger the volume of WMH, the more obvious the decline of the whole brain function or the cognitive performance defects in specific regions (after 1 month of onset).^[20]^ Thus, more studies should provide more information regarding CSVD patients to evaluate the value of MRI. Thus, 1 cross-sectionally investigation was conducted by Passiak BS and coworker.^[21]^ This study included 327 CSVD patients, collected the MRI data containing WMH, PVS, CMB, and cerebral atrophy, and the results sugessted, like other patients with cognitive impairment, increased WMH reflected the cognitive decline, WMH was also related to the decline in information processing speed. However, data on the relationship between PVS and cognitive impairment and dementia are very limited,^[22]^ and more studies are needed to confirm the relationship between PVS and cognitive impairment.

Besides, Nannoni S et al^[23]^ confirmed that CMB is strongly correlated with VCI, especially in terms of execution speed and processing speed. The finding that there was still a strong association between CMB and VCI after the number of lacunar infarcts and the severity of WMH were adjusted provides support for CMBS as an independent risk factor for cognitive impairment. Studies have shown that high CMB counts are associated with an increased risk of cognitive deterioration in patients with cerebrovascular disease^[24–26]^and in the general population.^[27–29]^ This situation may indicate the threshold effect of CMB load on cognitive function. The more lesions there are the more severe cognitive function decline. Brain atrophy is also closely related to cognitive decline related to CSVD.^[30]^

## 3. Gait impairment and CSVD

### 3.1. Overview of gait impairment

Compared with a healthy aging population, 19% of CSVD patients aged 50 to 85 years old have abnormal gait, and gait disorders are often accompanied by cognitive decline.^[31,32]^ Gait impairment increases the disability rate of patients.^[33]^ According to relevant studies, gait impairment in patients with CSVD is mainly reflected in reduced stride speed, reduced stride length^[34]^ and doubled time from sitting position to standing position.^[35]^ However, other studies have shown that gait impairment in patients with CSVD is mainly manifested as rhythm defects, while speed is basically not affected.^[36]^

### 3.2. Risk factors for gait impairment in CSVD

Among all the imaging markers of CSVD, WMH is most closely associated with abnormal gait. Studies have shown that the rapid progression of WMH is an important risk factor for lower limb function decline in the aging process of the elderly.^[37]^ WMH affects gait function in 2 ways. First, the influence of abnormal macrostructure (lesion volume and spatial location).^[38–41]^ Su N et al^[41]^ conducted a cross-sectional study on 770 community people (CSVD burden), showing that the quantitative volume of WMH, especially the WMH located around the ventricle, is correlated with impaired lower limb motor function, and gait can be manifested as slow walking speed and delayed postural control, however, this study did not exclude the condition of dementia, which limited the evaluation of the correlation between imaging markers and motor impairment. The second is the influence caused by the change of white matter microstructure.^[42–44]^ This possibility was verified further by Bedda L Rosario and colleagues, they collected the data of WMH, fractional anisotropy, and gait speed for CSVD patients, and found that there is an interactive effect between WMH and white matter microstructure integrity, and better white matter integrity can compensate for the negative effect of WMH on walking function.^[45]^

Nevertheless, the relationship between lacunae and CSVD-induced gait disorders remains uncertain. De laat^[46]^ found that lacunae were correlated with gait abnormalities. The results of Webster and Choi^[47,48]^ showed that lacunae is correlated with step speed, step length, step width and step frequency, etc, and space may increase the risk of falling. However, other studies have shown that lacunae and micro bleeding are not correlated with gait impairment.^[38,41]^

Meanwhile, Su Ning et al^[41]^ found that CSVD-related brain microstructure changes may affect the motor function of lower limbs and upper limbs, and WMH and brain atrophy are related to the deterioration of motor function. Kubota Megumi et al^[49]^ found that the expanded perivascular space in the white matter region of the brain was involved in the occurrence of CSVD dyskinesia. In addition, Patel P and Savica R both found^[50,51]^ that cognitive impairment is related to gait impairment and may affect each other.

## 4. Affective impairment and CSVD

### 4.1. Overview of affective impairment

CSVD patients are prone to hallucinations, agitation, depression, anxiety, disinhibition, apathy, irritability, sleep disorders, appetite changes, and other neuropsychiatric symptoms, among which the incidence of anxiety and depression is high, mainly manifested as apathy and vascular depression. These emotional disorders can magnify the physical symptoms of neurological impairment, aggravate the disorder of psychological regulation, further hinder the recovery of neurological function, reduce the ability to do daily living and affect the prognosis of patients. Negative emotions in patients with CSVD will not only hinder the recovery of the patient’s condition but also aggravate the patient’s condition and prolong the patient’s hospital stay.

### 4.2. Risk factors for affective impairment in CSVD

The depressive symptom is one of the affective impairment for CSVD patients, Van Sloten TT et al^[52]^ followed up with 1949 participants for 5 years and found that various markers of CSVD (imaging), including included WMH volume, subcortical infarcts, cerebral microbleeds, Virchow-Robin spaces and total brain parenchyma volume, were highly correlated with the incidence of depressive symptoms, and CSVD located deep in the brain had a higher incidence of depressive symptoms than other brain regions. This is the clearest evidence to date that CSVD is a risk factor for depression and imaging markers may assist the diagnosis of the affective impairment.

Intriguingly, Van Uden IW^[53]^ also found that the integrity of the white matter tract in the elderly with depression was lower, independent of the overall cognitive function, and the existence of CSVD was the main reason. Furthermore, to investigate the to investigate baseline predictors of depression of CSVD, 1 study of Pavlovic AM^[54]^ showed that WMH lesion degree and cognitive impairment could predict the occurrence of delayed depressive disorder in CSVD patients. Besides, Direk N et al^[55]^ also observed similar findings that greater WMH volume was positively correlated with the severity of depression in the community population. In addition, Heiden A et al^[56]^ followed up with patients with leukoencephalopathy and found that compared with patients without leukoencephalopathy, patients with leukoencephalopathy had more severe depressive disorder, and the severity of leukoencephalopathy was positively correlated with the Hamilton depression rating scale score, suggesting that WMH are related to poorer prognosis of elderly depressed patients, but it is still needed more study to research WHMs as a prognostic factor for therapeutic decision-making. Moreover, Aizenstein HJ et al^[57]^ found that the integrity and physiological function of white matter nerve fibers in the frontal, temporal, and midbrain of elderly patients with depressive disorder were damaged, and the integrity of nerve fibers in the limbic orbitofrontal network was damaged during the course of the disease.

Besides, research shows that CMB not only induces cognitive impairment, but also leads to emotional impairment, which may be because CMB damages brain tissue, leads to the damage of cortico-subcortical fiber and white matter conduction pathway, and then induces affective impairment.^[58]^ Kim^[59]^ found that patients with leukoencephalopathy had more serious anxiety and depression than healthy people, and the more severe the degree of leukoencephalopathy was, the higher the HAMA and Hamilton depression rating scale scores were.

## 5. Urination disorder and CSVD

### 5.1. Overview of urination disorder

Urination disorder is considered to be one of the main clinical manifestations of CSVD, which seriously affects the daily life and social function of CSVD patients, including frequent urination, urgency of urination, incontinence, nocturia, etc. At present, the incidence of urination disorder in elderly people over 65 years old has reached 75%.^[60,61]^ Urination disorder is a more common but easily ignored symptom in clinical practice. Compared with other symptoms, the research on urination disorder in CSVD patients is still far behind. In cerebrovascular diseases, the most common urinary symptoms are increased nocturia and urge incontinence caused by overactivity of the detrusor muscle,^[62]^ which can be manifested as frequent urination in the early stage of the disease, urinary incontinence in the middle stage, and complete urinary incontinence in the end stage of the disease.^[1,63]^ Studies have found that urination disorders may appear earlier than cognitive dysfunction and gait changes.^[64]^

### 5.2. Risk factors for urination disorder in CSVD

Given known studyies, WMH is associated with urinary disorders (such as frequent urination and incontinence) in cerebrovascular diseases.^[65]^ Further analysis of the correlation between the severity of WMH, the number of lacunar cerebral infarctions and urinary symptoms showed that patients with severe WMH had more frequent urination urgency, and patients with severe WMH had a 74% higher risk of urge urinary incontinence than patients with mild WMH, while patients with lacunar cerebral infarction had a higher prevalence of urinary incontinence and urinary frequency.^[66]^ To analyze that involvement of areas critical to bladder control could influence urinary incontinence, Kuchel GA et al^[67]^ studied the relationship between leukodystrophy sites and voicing disorders and found that leukodystrophy in the right frontal lobe and right sub-frontal region may be related to the severity of urinary incontinence. It was found that WMH could explain and predict dysuria in the elderly population.^[68]^ Besides, Emergency urination was independently associated with severe WMH after controlling for age, sex, space between cavities, diabetes, and diuretic use.^[66]^ Similarly, WMH was significantly associated with the urgency of urination in Alzheimer patients.^[69,70]^ However, no independent relationship has been found between lacunae and voiding disorders. Neither the presence nor the number of lacunae has been found to be significantly correlated with voiding disorders.^[66]^

## 6. Swallowing disorder and CSVD

### 6.1. Overview of swallowing disorder

Dysphagia is a disorder in the intake and transportation of food from the mouth through the pharynx and esophagus to the stomach, resulting in an inability to eat safely and efficiently to obtain adequate nutrition and hydration. Dysphagia can cause a variety of complications, such as pneumonia, dehydration, malnutrition, etc, which can directly or indirectly affect the long-term prognosis and quality of life of patients. The appearance of CSVD can lead to dysphagia, but it has been poorly reported.

### 6.2. Risk factors for swallowing disorder in CSVD

Maeshima et al^[71]^ studied the relationship between cerebral microbleeds and dysphagia using low-density shadow (containing hemosiderin) of T2-weighted imaging. The results showed that cerebral microbleeds were significantly correlated with dysphagia, especially when both sides had microbleeds. A retrospective study found that high white matter signals affected the swallowing function of elderly patients with mild stroke, and the severity of white matter lesions could be used as a predictor of swallowing disorder.^[72]^

## 7. Early diagnosis via MRI method

## 8. Discussion

With the aging of the population, the incidence of CSVD is on the rise, which is a concern for the public health system. The most common symptoms of CSVD are cognitive dysfunction, movement disorders, emotional disorders, voiding disorders and decreased social ability. These symptoms seriously affect the quality of life of patients and bring a heavy burden to patients families and society. However, there are still many unknown areas in the current research on CSVD, such as the occurrence and development of the disease, the relationship between CSVD and related clinical manifestations is still not completely clear, especially for the imaging markers of CSVD and the evaluation criteria of its overall load. Besides, CSVD is a clinical, neuroimaging and pathological syndrome of the whole brain, also with diverse complications, therefore, early diagnosis is crucial for disease control and treatment. However, only 1 imaging marker may not be sufficient for the disease early diagnosis. The Scoring system of baseline CSVD load proposed by the collaborative group of Maastricht University in the Netherlands in 2013 has been widely used in clinical studies at home and abroad as a classic evaluation method,^[73]^ which avoided excessive dependence on a single image marker and the influence of confounding factors, and improved the early diagnosis rate. In addition, MRI marker is essential, but other clinical test is also necessary for auxiliary diagnosis of CSVD. Moreover, there are relatively few effective treatment options for CSVD at present, and most of them focus on prevention, for example, blood pressure control, emotion regulation, rational diet, and motor cognitive training. Therefore, early assessment of CSVD patients, early detection of risk factors, and timely intervention are beneficial to delay the decline of patients’ daily living ability, improve the quality of life of patients, and reduce the psychological burden of patients and their families. For a deeper understanding of the disease, clinical and basic workers still need to continue to carry out a large number of experimental observations and research.

## Acknowledgments

The authors thank Professor Zhirong Liu for his kind advice on this article.

## Author contributions

**Supervision:** Zhirong Liu.

**Writing – review & editing:** Yang Wang.
